# Mechanical Properties and Characterization of Epoxy Composites Containing Highly Entangled As-Received and Acid Treated Carbon Nanotubes

**DOI:** 10.3390/nano11092445

**Published:** 2021-09-19

**Authors:** Aaron S. Krieg, Julia A. King, Gregory M. Odegard, Timothy R. Leftwich, Leif K. Odegard, Paul D. Fraley, Ibrahim Miskioglu, Claire Jolowsky, Matthew Lundblad, Jin Gyu Park, Richard Liang

**Affiliations:** 1 Michigan Technological University, 1400 Townsend Drive, Houghton, MI 49931, USA; askrieg@mtu.edu (A.S.K.); jaking@mtu.edu (J.A.K.); trleftwi@mtu.edu (T.R.L.); lkodegar@mtu.edu (L.K.O.); pdfraley@mtu.edu (P.D.F.); imiski@mtu.edu (I.M.); 2 Florida State University, High-Performance Materials Institute, 2005 Levy Ave, Tallahassee, FL 32310, USA; cj16d@my.fsu.edu (C.J.); ml19r@my.fsu.edu (M.L.); parkji@eng.famu.fsu.edu (J.G.P.); liang@eng.fsu.edu (R.L.)

**Keywords:** multi-walled carbon nanotubes, epoxy composites, flexural strength, functionalized nanotubes

## Abstract

Huntsman–Merrimack MIRALON^®^ carbon nanotubes (CNTs) are a novel, highly entangled, commercially available, and scalable format of nanotubes. As-received and acid-treated CNTs were added to aerospace grade epoxy (CYCOM^®^ 977-3), and the composites were characterized. The epoxy resin is expected to infiltrate the network of the CNTs and could improve mechanical properties. Epoxy composites were tested for flexural and viscoelastic properties and the as-received and acid treated CNTs were characterized using Field-Emission Scanning and Transmission Electron Microscopy, X-Ray Photoelectron Spectroscopy, and Thermogravimetric Analysis. Composites containing 0.4 wt% as-received CNTs showed an increase in flexural strength, from 136.9 MPa for neat epoxy to 147.5 MPa. In addition, the flexural modulus increased from 3.88 GPa for the neat epoxy to 4.24 GPa and 4.49 GPa for the 2.0 wt% and 3.0 wt% as-received CNT/epoxy composites, respectively. FE-SEM micrographs indicated good dispersion of the CNTs in the as-received CNT/epoxy composites and the 10 M nitric acid 6 h treatment at 120 °C CNT/epoxy composites. CNTs treated with 10 M nitric acid for 6 h at 120 °C added oxygen containing functional groups (C–O, C=O, and O=C–O) and removed iron catalyst present on the as-received CNTs, but the flexural properties were not improved compared to the as-received CNT/epoxy composites.

## 1. Introduction

The carbon nanotube (CNT) is an ultra-strong and ultra-stiff material due to its covalent sp^2^ bonds formed between carbon atoms (Elastic modulus [E] = 1 TPa, tensile strength [σ] = 50–500 GPa) [1,2,3]. Due to these outstanding properties, CNTs have been added to many composite materials. However, the extremely high CNT tensile strength and modulus have not been translated into corresponding high composite properties [4,5,6,7,8]. Novel bulk formats of CNT assemblages, such as sheets, tapes, and yarns, have more recently been made available which could be used for manufacturing macro-scale CNT composites for aerospace applications. MIRALON^®^ pulp is a novel and unique format of CNTs that are manufactured from a floating catalyst chemical vapor deposition (FC-CVD) reactor. MIRALON^®^ CNTs are networked, highly entangled, commercially available, and scalable with very high aspect ratios. Due to the scalability of these unique CNTs, there is considerable interest from the aerospace community in their use on a commercial scale. These highly networked CNTs are anticipated to infiltrate the aerospace grade toughened epoxy resin which could improve mechanical properties.

The realization of the reinforcing effect of high strength/modulus nanotubes relies on the ability to transfer load between epoxy and nanotube phases of the composite, due to van der Waals forces or, in the case of some functionalized nanotubes, covalent linkages. Many methods for promoting covalent functionalization of the CNT have been explored in the literature (e.g., oxygenation, amination, fluorination, etc.) [9,10,11,12]. This paper focuses on a nitric or nitric/sulfuric acid treatment, which is a mechanism that is well established in the literature for the introduction of oxygen containing functional groups onto the CNT surface [13,14,15]. The mechanism of oxygenation of CNTs by means of acid treatment typically occurs stepwise by generation of C-O and C=O groups at defect sites which are further transformed into COOH groups in highly acidic environments [16,17,18].

In this work, researchers fabricated aerospace grade epoxy (CYCOM^®^ 977-3) composites containing various amounts of as-received CNTs (MIRALON^®^ pulp) and acid treated CNTs. The CYCOM^®^ 977-3 resin is a high-performance aerospace grade resin toughened with a thermoplastic co-polymer and is used in state-of-the-art aerospace polymer composite structures. Composites were tested for flexural properties as well as dynamic mechanical analysis (DMA) to study the compatibility of the matrix-filler material and changes in glass transition temperature, respectively. In addition, MIRALON^®^ CNT pulp was treated in a variety of acid treatment methods and characterized for surface morphology, graphitic character, surface chemistry, and thermal stability using Field Emission Scanning Electron Microscopy (FE-SEM), Transmission Electron Microscopy (TEM), Raman spectroscopy, X-Ray Photoelectron Spectroscopy (XPS), and Thermogravimetric Analysis (TGA). Per the authors knowledge, these material properties have never been previously reported in the open literature.

## 2. Materials and Methods

### 2.1. Materials

The epoxy matrix used was Solvay CYCOM^®^ 977-3 epoxy resin (Solvay, Brussels, Belgium). The CYCOM^®^ 977-3 resin is a high-performance toughened epoxy system with a proprietary blend of epoxy and a thermoplastic copolymer with a co-continuous morphology. When ramping 2 °C/min, the viscosity of the resin decreases to a minimum of 600 cP at 155 °C and begins climbing again at 170 °C due to a crosslinking reaction. The density of the cured resin at 25 °C is 1.29 g/cm^−3^ [19].

The CNT filler was a Huntsman–Merrimack MIRALON^®^ CNT pulp (Huntsman, Merrimack, NH, USA). The MIRALON^®^ pulp is manufactured by chopping non-woven MIRALON^®^ CNT sheets into small porous bundles that are approximately 0.05 mm (50 µm) in diameter and 1 mm in length. CNTs are unraveled when dispersed in resin, with individual fiber diameters of 0.01 µm (10 nm). The MIRALON^®^ pulp used was manufactured using high growth rate (HGR) grade MIRALON^®^ CNTs. HGR MIRALON^®^ pulp contains approximately 30 wt% iron carbide and a 5 wt% amorphous carbon content [20]. The stock reagents used to functionalize the carbon nanotubes were 98 w/w% sulfuric acid (Fisher Scientific, Hampton, NH, USA) and 75 w/w% nitric acid (VWR International, Radnor, PA, USA).

### 2.2. CNT Acid Treatment Methods

#### 2.2.1. Nitric/Sulfuric Acid Treatment at 23 °C

MIRALON^®^ pulp was treated with a sulfuric/nitric acid mixture (0.5 L) in a 1 L glass Erlenmeyer flask. The acid mixture was 3:1 (by volume) of 10.0 M sulfuric acid (0.33 L) and 4.0 M nitric acid (0.17 L), respectively. CNT pulp (2.5 g) was added to the acid solution and the CNT suspension was mixed at 23 °C with a PTFE magnetic stir bar (13 mm diameter and 76 mm long) on a magnetic stir plate, for either 6, 12, or 18 h.

The CNTs were separated using a vacuum filtration assembly with a 55 mm diameter filter with 2.5 µm pore size (Whatman Grade 42). Iterations of washing the CNTs with water in the vacuum filtration assembly were performed until pH probe detected a near-neutral pH (between 6 and 7). The CNT cake was retrieved from the filtration apparatus and dispersed in methylethylketone (MEK) (Fisher Scientific, Hampton, NH, USA). The suspension was poured into glass petri dishes and placed in an oven at 95 kPa vacuum at 23 °C for 3 h with a liquid nitrogen cold trap to collect the MEK. CNTs were retrieved from the bottom of the petri dishes. CNTs were then placed in an oven to dry for 6 h at 120 °C at 95 kPa vacuum. After drying, the acid treated pulp was stored in a moisture barrier bag.

#### 2.2.2. Nitric Acid Treatment at 90 °C and 120 °C

MIRALON^®^ pulp was treated with aqueous nitric acid at 2.5 M and 10.0 M concentrations (0.5 L) in a 1 L glass Erlenmeyer flask. CNT pulp (2.5 g) was added to the solution, and the CNT suspension was sonicated for 30 min in a Branson sonicator (Branson Ultrasonics Corporation, Brookfield, CT, USA) at 40 Hz and refluxed for 3 h at 90 °C or for 6 h at 120 °C. The same method was used to process the treated CNTs as was described in the nitric/sulfuric acid treatment discussed above in Section 2.2.1.

### 2.3. Specimen Fabrication

**Figure 1:**
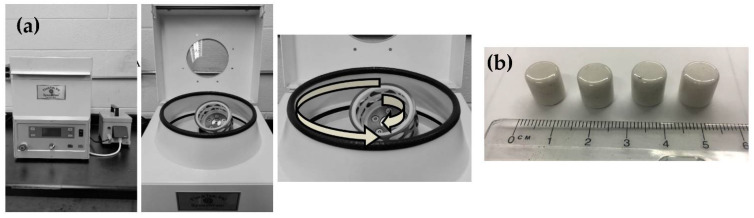
(**a**) FlackTek SpeedMixer DAC 150.1 FVZ; (**b**) Zirconium cylinders used for mixing media in SpeedMixer.

Plaques of CYCOM^®^ 977-3 epoxy were fabricated by mixing epoxy in a FlackTek SpeedMixer DAC 150.1 FVZ (FlackTek, Inc., Landrum, SC, USA), which is a planetary centrifugal mixer and is shown in Figure 1. Each of 2 SpeedMixer cups (100 g capacity) were charged with 70 g of neat epoxy resin. The cups of resin were heated to 100 °C in a Fisher Isotemp 282A (Fisher Scientific, Hampton, NH, USA) vacuum oven and held for 30 min at atmospheric pressure. Then, the cups of resin were mixed in the SpeedMixer at 100 °C for 1 min at 1750 rpm, 2 min at 2500 rpm, and 1 min at 3000 rpm. Then the cups were placed back in the oven at 100 °C and at atmospheric pressure for 10 min. Three zirconium mixing cylinders (1 cm diameter, 1 cm height) were added to each cup, and the mixing cycle (1 min at 1750 rpm, 2 min at 2500 rpm, and 1 min at 3000 rpm) was repeated once.

Neat resin was then degassed in the vacuum oven at 100 °C and held for 10 min at 101 kPa vacuum. The resin was degassed for 30 min at 100 °C by alternating between atmospheric pressure and 101 kPa vacuum. Then, 101 kPa vacuum was applied to a vacuum chamber for an additional 2 h. Then, the resin was removed from the oven to cool at 23 °C and was then placed in the freezer at −18 °C until the plaques were manufactured.

Neat epoxy plaques were manufactured using a Wabash Vantage Series Model V75H-18-CLX (Wabash MPI Carver, Wabash, IN, USA) compression molding press. All steel from the tooling assembly was sprayed with Mann Ease 2300 (Mann Release Technologies, Macungie, PA, USA) release compound. Frozen pieces of degassed resin were placed in a 20 cm × 20 cm steel chase that sat on the bottom half of the steel assembly. The steel chase generated 15 cm × 25 cm plaques that are 3.2 mm thick. The tooling assembly was vacuum bagged at 101 kPa vacuum and placed in the compression molding machine and pressed with 10 kN force. The compression molding machine was heated to 100 °C at 101 kPa vacuum pressure and held for 20 min. Platens were then ramped to 130 °C and held for 30 min at 130 °C. The platen temperature was then ramped to 177 °C, and 25 kN was applied and held at 177 °C for 6 h. Platens were cooled with air and water from 177 °C to 150 °C. Cooling air was turned off, and water cooling continued from 150 °C to 30 °C. The pressure was released, and the assembly was removed. Test samples were cut from the plaque.

Plaques of as-received CNT/epoxy were fabricated by mixing MIRALON^®^ pulp with epoxy in a SpeedMixer to achieve target CNT concentrations (0.4 wt%, 0.8 wt%, 1.5 wt%, 2.0 wt%, and 3.0 wt%). Each of 2 SpeedMixer cups were charged with 70 g of neat 977-3 resin. The appropriate amount of MIRALON^®^ pulp was added to each cup to produce the target formulation of CNT/epoxy. The same method was used to mix, degas, and compression mold as-received CNT/epoxy composite plaques as was described above for the neat epoxy plaques.

Plaques of epoxy composites made with CNTs treated with nitric/sulfuric acid mixture at 23 °C and with 2.5 M nitric acid at 90 °C were fabricated using the same method as described for as-received CNT/epoxy composites described above. Plaques of epoxy composites made with CNT pulp treated with 10.0 M nitric acid at 120 °C were fabricated by sonicating 30 g of MEK and 0.28 g of treated CNTs in each of two test tubes in the Branson sonicator for 2 h at 40 Hz. Then 70 g of epoxy resin was combined with the MEK/CNT suspension in each of 2 SpeedMixer cups and mixed in the SpeedMixer at 23 °C for 10 min at 1750 rpm and 2 min at 2500 rpm. The mixture was put in an oven at 100 °C and 101 kPa of vacuum for 1 h with a liquid nitrogen cold trap to collect the MEK. Three zirconium mixing cylinders (1 cm diameter, 1 cm height) were added to each cup, and the mixing cycle (1 min at 1750 rpm, 2 min at 2500 rpm, and 1 min at 3000 rpm) was repeated once at 100 °C. The same method was used to degas and compression-mold the acid treated CNT/epoxy composites as was described above for the neat epoxy plaques.

### 2.4. Flexural 3-Point Bend Test Method

Flexural properties were obtained using a 3-point bend testing method at 23 °C on neat CYCOM^®^ 977-3 epoxy plaques and MIRALON^®^ CNT/epoxy plaques. ASTM D790 3-point bend test method was used with an Instron 4206 screw driven mechanical testing machine (Instron, Norwood, MA, USA) [21]. Specimens were supported on 5 mm diameter support pins and loaded at mid-span using a 5 mm diameter loading roller pushed down by the mechanical testing frame. Specimens were tested with a 16:1 support span to thickness ratio (support span = 51.2 mm, thickness = 3.2 mm) and a crosshead speed of 5.3 mm/min. Sample dimensions were 12.7 mm wide and 7.2 cm long. Samples were conditioned at 50% relative humidity at 23 °C for 40 h before testing. A 1.3 kN load cell was used to measure loading force, and a linear variable displacement transducer (LVDT) was used to measure deflection. The flexural modulus was calculated from the initial linear portion of the stress–strain curve. The flexural strength and strain were determined at the highest stress recorded before failure. For each formulation, at least 7 samples were tested.

### 2.5. Dynamic Mechanical Analysis (DMA) Test Method

Glass transition temperatures (T_g_) for neat epoxy and CNT/epoxy composites were obtained according to ASTM D7028 using a TA Instruments Q800 DMA (TA Instruments, New Castle, DE) with a single cantilever clamp [22]. DMA specimens were 35 mm long, 12.8 mm wide, and 3.2 mm thick. An oscillating amplitude of 30 µm at 1 Hz constant frequency was applied to the specimen with an initial temperature of 50 °C and a soak time of 5 min before temperature ramping at a rate of 3 °C/min to a temperature that was 40 °C higher than the T_g_ for the material (150 °C). The sample was then air cooled to 30 °C.

To perform the glass transition temperature (T_g_) tests, two temperature ramps were performed for each specimen. The first temperature ramp was to fully cure the resin system and to erase the thermal history of the sample. A second temperature ramp was used to locate the T_g_. Once the test was completed, the data were analyzed to determine the T_g_ by plotting the tanδ curve against temperature. The T_g_ was determined to be at the peak of the tanδ curve.

### 2.6. Field Emission Scanning Electron Microscope (FE-SEM) Test Method

A Hitachi S-4700 field emission scanning electron microscope (FE-SEM) (Hitachi High-Technologies Corporation, Tokyo, Japan) was used to view as-received and acid treated MIRALON^®^ CNT pulp. To obtain images of the bulk CNT, MIRALON^®^ pulp was placed on carbon tape on aluminum sample studs. To obtain micrographs of individual CNT bundles, 5 mg of CNTs were sonicated in 20 mL of ethanol for 30 min, and 2 drops were placed on aluminum sample studs and left under a fume hood for 12 h. Micrographs were collected using 10 kV accelerating voltage and 5 µA emission current. The upper secondary electron detector was used to collect the images.

The FE-SEM was used to view the flexural fracture surfaces of CNT/epoxy composite samples. The samples were cut to 3.2 mm × 2 mm × 13 mm (thickness × width × length) and placed on carbon tape on an aluminum sample stud. Then, the samples were sputter coated with a 2-nm thick platinum/palladium coating using a Cressington 208HR sputter coater. The film thickness was measured with a Cressington MTM-20 High Resolution Thickness Controller (Cressington Scientific Instruments, Watford, UK). Samples were viewed using 10 kV accelerating voltage and 10 µA emission current. The upper secondary electron detector was used to collect the images.

### 2.7. Transmission Electron Microscope (TEM) Test Method

As-received and acid treated MIRALON^®^ CNT pulp were dispersed in isopropanol using an ultrasonic homogenizer (Misonix Inc., Farmingdale, NY, USA) for 2 min. Dispersed solution was dropped on the Lacey carbon grid and dried overnight. CNT structures were observed at high resolution transmission electron microscope (JEM-ARM200cF, JEOL USA, Peabody, MA, USA) with 80 kV acceleration voltage.

### 2.8. Thermogravimetric Analysis (TGA) Test Method

A TA Instruments Q50 thermogravimetric analyzer (TA Instruments, New Castle, DE) was used to conduct thermal analysis experiments of the as-received and treated CNT pulp per ASTM E2550 to determine the residual catalyst wt% and to detect changes in weight [23]. In addition, 40 mL/min of nitrogen was used as a protective gas, and 60 mL/min of air was used as a reactive gas. The heating rate used was 10 °C/min from 25 °C to 900 °C. An initial 4 mg sample was placed on a platinum pan. Universal analysis software from TA Instruments was used to analyze the resulting data.

### 2.9. Raman Spectroscopy Test Method

The Raman spectrographs of the nanotube pulp samples were taken with a Reinshaw inVia Raman microscope system (Renishaw Inc., Wotton-under-Edge, UK). A 785 nm laser was used to analyze a spectral range from 897 to 1955 shift/cm^−1^. The laser was calibrated using an internal silicon standard before data were taken. Laser power was set at 1% with an exposure time of 20 s. The measurement was generated and analyzed using WiRE3 software.

### 2.10. X-ray Photon Spectroscopy (XPS) Test Method

The XPS instrument used was a Phi 5800 XPS (Physical Electronics, Chanhassen, MN, USA) equipped with a dual source (Mg/Al) anode for generating X-rays. The nanotube samples for XPS were placed on a 25 mm diameter sample puck. Sheet samples were cut to 10 mm^2^ squares and held on to the sample puck with a spring clip. Powder samples were placed on a piece of double-sided copper tape that was adhered to the puck. The samples were placed in a transfer chamber that was pumped down to high vacuum prior to being transferred into the ultra-high vacuum (UHV) analysis chamber, with a base pressure better than 1 × 10^−7^ Pa. Mg X-rays (photon energy = 1254.6 eV) were used to analyze the nanotube samples. The X-ray power was 400 W for sheets and 100 W for the powder samples.

The XPS survey spectra were collected using a pass energy of 187.85 eV, a data spacing of 0.8 eV/step, a dwell time of 20 ms/step and a range from 0 to 1175 eV. The survey spectra were collected for 5 min (10 scans). The high-resolution C1s spectra were collected using a pass energy of 23.50 eV, a data spacing of 0.1 eV/step, and a dwell time of 100 ms/step. The high-resolution C1s spectra were collected for 5 min (15 scans).

## 3. Results

### 3.1. Flexural 3-Point Bend Results

Table 1 shows the flexural results for neat epoxy and as received CNT/epoxy composites. This table shows that our neat epoxy modulus and strength (3.88 ± 0.16 MPa and 136.9 ± 5.0 MPa, respectively) fall within the ranges reported by vendor literature [19]. All these results were compared at the 95% confidence level. The neat epoxy and the CNT composites containing up to 1.5 wt% have the same flexural modulus. The flexural modulus is statistically significantly higher for the 2.0 wt% CNT/epoxy (4.24 GPa) as compared to our neat epoxy composites (3.88 GPa). For the 3.0 wt% CNT/epoxy composite (4.49 GPa), the flexural modulus is statistically significantly higher than the neat and the 2.0 wt% CNT/epoxy composite. For the 0.4 wt% CNT/epoxy composite, the flexural strength (147.5 MPa) is significantly higher than for the neat epoxy (136.9 MPa). For the composites containing 0.8 to 2.0 wt% CNT, the flexural strengths are the same. For the 3.0 wt% CNT/epoxy composite, the flexural strength (116.4 MPa) is significantly lower than the neat epoxy. The flexural strain is statistically the same for all composites containing ≤2.0 wt% CNT. For the 3.0 wt% CNT/epoxy composite, the flexural strain is statistically lower than the neat epoxy.

Jeong Tai et al. [24] reported an increase in flexural modulus from 2.10 GPa to 2.35 GPa and an increase in flexural strength from 67 MPa to 75 MPa when dispersing 0.5 wt% as-received MWCNTs in a diglycidyl ether of bisphenol A (DGEBA) type epoxy resin (Kukdo Chemical YD-115) with a polyamidoamine hardener (Kukdow Chemical G-A0533) using an ethanol solvent and a hotplate stirrer. Garg et al. [25] reported an increase in flexural modulus from 2.35 GPa to 3.0 GPa when dispersing 0.5 wt% as-produced MWCNTs in a DGEBA type epoxy resin (Huntsman LY-556) with an aliphatic amine hardener (Huntsman Aradur HY 951) using an acetone solvent and a hotplate stirrer. In our work, we noted an increase in flexural modulus for composites containing ≥2.0 wt% CNT. Garg et al. also reported an increase in ultimate flexural strength from 55 MPa to 90 MPa at 0.3 wt% concentration of as-produced MWCNTs before declining with further addition of CNTs. This initial flexural strength increase followed by a decline at higher CNT concentrations agrees with our work.

Since the flexural strength increased for the 0.4 wt% CNT/epoxy composite, this formulation was investigated further by various acid treatments. It is possible that adding additional oxygen containing functional groups to the CNT surface could increase CNT/epoxy composite flexural properties. Table 2 shows the flexural results for various acid treated 0.4 wt% CNT/epoxy composites along with neat epoxy and as received 0.4 wt% CNT/epoxy formulations. The data in Table 2 shows that in all cases, the acid treated CNT/epoxy composites did not improve the flexural properties.

### 3.2. DMA Results

The glass transition temperature (T_g_) is the temperature during heating at which an amorphous solid becomes soft as it transitions from a glassy state to a rubbery state. Glass transition temperatures (T_g_) of neat 977-3 and as-received MIRALON^®^ CNTs/977-3 composites are shown in Table 3. The tanδ T_g_ for neat 977-3 was found to be 249.55 ± 0.60 °C. At the 95% confidence level, there is no statistical difference between the neat epoxy and ≤2.0 wt% CNT/epoxy composites. Seyler [26] reported a tanδ T_g_ of 261 °C using DMA testing on neat 977-3 epoxy specimens while testing at 1 Hz frequency and ramp rate of 10 °C/min. Koo et al. [27] reported a tanδ T_g_ of 237 °C using DMA testing on neat 977-3 epoxy specimens while testing at 1 Hz frequency and ramp rate of 5 ° C/min. Our neat epoxy T_g_ is between those of these two researchers. Valentini et al. and Kumar et al. [28,29] have also reported no change in T_g_ for composites containing ≤0.2 wt% CNT in epoxy.

### 3.3. FE-SEM Results

**Figure 2:**
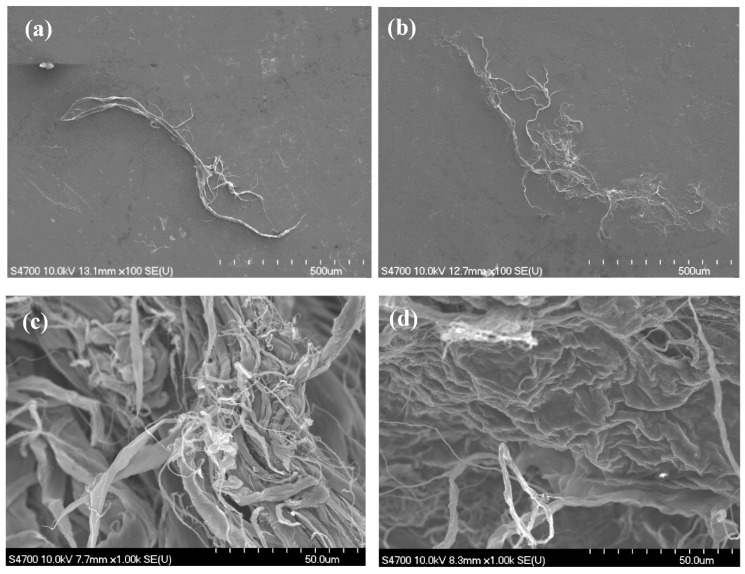
(**a**) FE-SEM image × 100 magnification for isolated bundle of as-received MIRALON® pulp; (**b**) FE-SEM image × 100 magnification for isolated bundle of MIRALON® pulp acid treated for 6 h in 10 M nitric at 120 °C; (**c**) FE-SEM image × 1000 magnification of entangled network for as-received MIRALON® pulp; (**d**) FE-SEM image × 1000 magnification of entangled network for MIRALON® pulp acid treated for 6 h in 10 M nitric at 120 °C.

**Figure 3:**
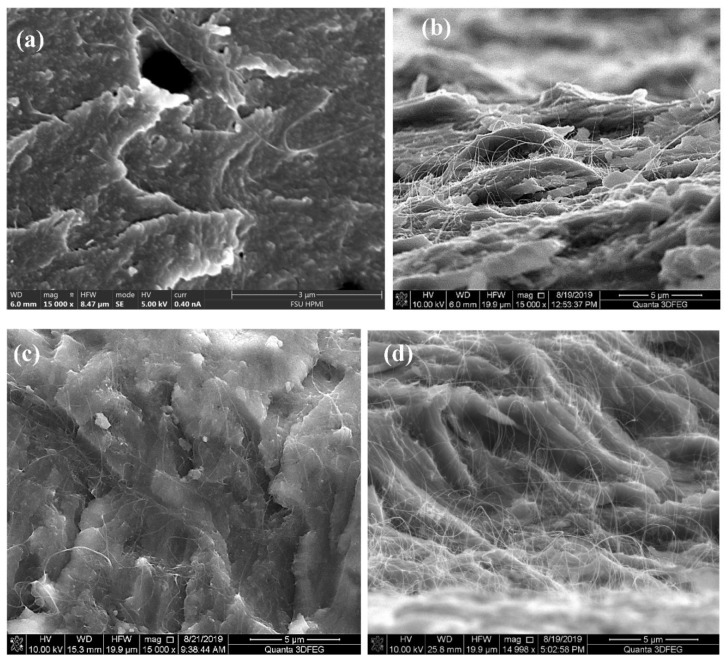
FE-SEM images ×15,000 magnification of flexural fracture surface of (**a**) neat epoxy; (**b**) 0.4 wt% as-received MIRALON^®^ pulp/epoxy; (**c**) 0.8 wt% as-received MIRALON^®^ pulp/epoxy; (**d**) 1.5 wt% as-received MIRALON^®^ pulp/epoxy.

**Figure 4:**
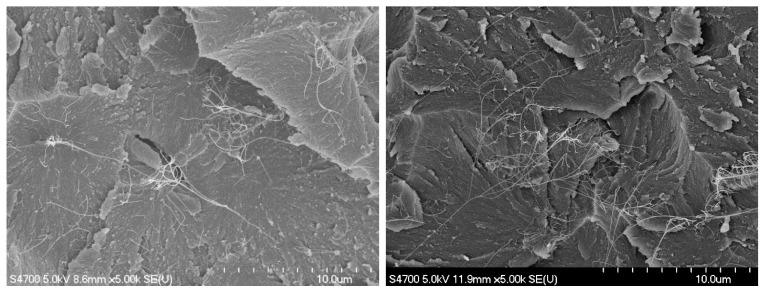
FE-SEM image ×5000 magnification of flexural fracture surface of 0.4 wt% as-received MIRALON^®^ pulp/epoxy (**left**) and 0.4 wt% MIRALON^®^ pulp acid treated (6 h in 10 M nitric at 120 °C)/epoxy (**right**).

Figure 2 shows FE-SEM images of the as-received and acid treated (6 h in 10 M nitric at 120 °C) MIRALON^®^ CNT pulp. In both cases, the isolated bundle diameters appear to be ≤50 µm and ~1 mm long. The acid treated pulp bundle appears to be more dispersed (spread out in the plane) than the as-received CNT pulp. Figure 2 also shows FE-SEM images of the entangled network before and after acid treatment (6 h in 10 M nitric at 120 °C). The acid treated network appears to be much more densified than the as-received CNTs. Figure 3 shows the flexural fracture surfaces of neat epoxy, 0.4 wt%, 0.8 wt%, and 1.5 wt% as-received MIRALON^®^ CNT pulp/epoxy. Good dispersion is observed in all epoxy composites containing up to 1.5 wt% CNTs. Figure 4 shows the flexural fracture surfaces of 0.4 wt% as-received MIRALON^®^ CNT pulp/epoxy and 0.4 wt% acid treated pulp (6 h in 10 M nitric at 120 °C)/epoxy. In both cases, it appears that there is good dispersion of CNT in the composite flexural fracture surface.

### 3.4. TEM Results

**Figure 5:**
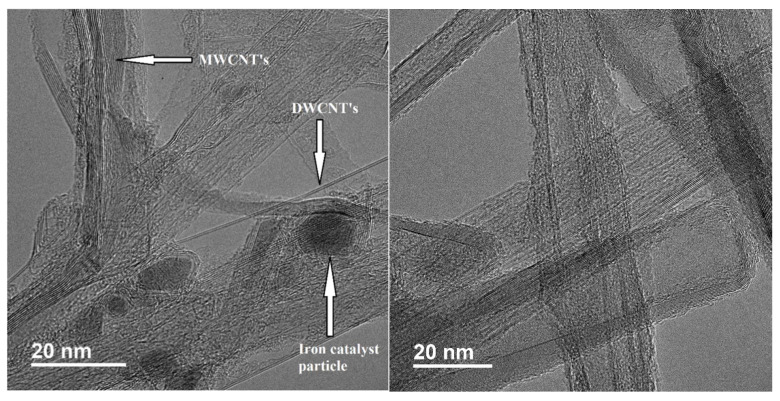
TEM images of as-received MIRALON^®^ pulp (**left**) and MIRALON^®^ pulp acid treated for 6 h in 10 M nitric acid at 120 °C (**right**).

Figure 5 shows TEM images of as-received MIRALON^®^ pulp and MIRALON^®^ pulp acid treated for 6 h in 10 M nitric acid at 120 °C. The images show double and multi walled CNTs with 10–20 nm diameters. Figure 3 shows some CNTs with 8 or more walls. Iron catalyst was seen in graphitic “cages” in the as-received CNT. Figure 5 also illustrates that the graphitic cages that once held the iron catalyst have been emptied by perforation of the hot nitric acid and subsequent dissolution of the metallic iron nanoparticles [30,31].

### 3.5. TGA Results

**Figure 6:**
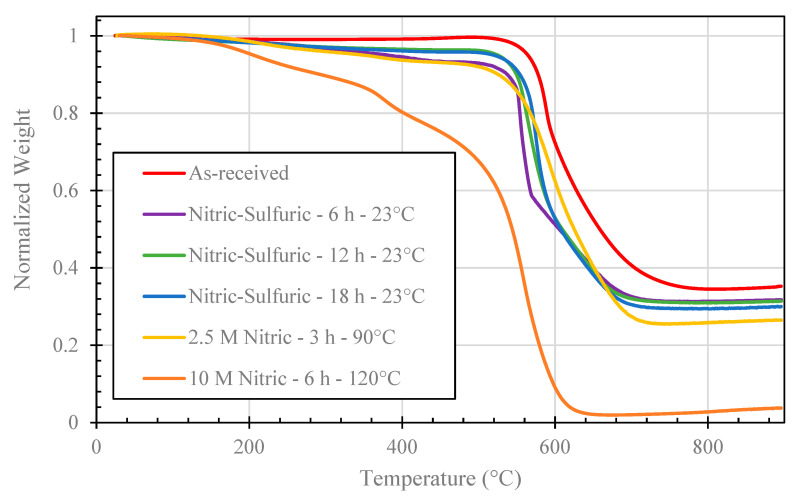
Normalized mass loss TGA results of CNT samples: as-received, treated with nitric/sulfuric acid mixture at 23 °C (6, 12, and 18 h), 2.5 M nitric acid at 90 °C for 3 h, and 10 M nitric acid at 120 °C for 6 h.

Figure 6 displays normalized weight of MIRALON^®^ CNTs plotted against temperature. Figure 4 shows that for as-received CNTs, the CNT wall oxidizes at 575 °C. For all the other curves, the mass loss in the region from 25 °C to 150 °C is related to the release of physically adsorbed water. For CNTs treated with nitric/sulfuric acid mixture at 23 °C (6, 12, and 18 h) and for CNTs treated with 2.5 M nitric at 90 °C for 3 h, there is 6% weight loss between 150 °C and 575 °C, likely due to decomposition of oxygen containing functional groups. The CNT treated for 6 h in 10 M nitric acid at 120 °C (most aggressive acidic treatment) showed the largest mass loss between 150 °C and 575 °C, which could be due to this sample containing the most oxygen containing functional groups. Moreover, the normalized weight at the final temperature for this sample was much less than that of the other CNTs studied.

Table 4 shows the iron content for each sample studied. The iron content is related to the normalized weight for each sample at the final temperature. The significant reduction in iron for the most aggressive acid treatment is consistent with the TEM photomicrograph shown previously.

### 3.6. Raman Spectroscopy Results

**Figure 7:**
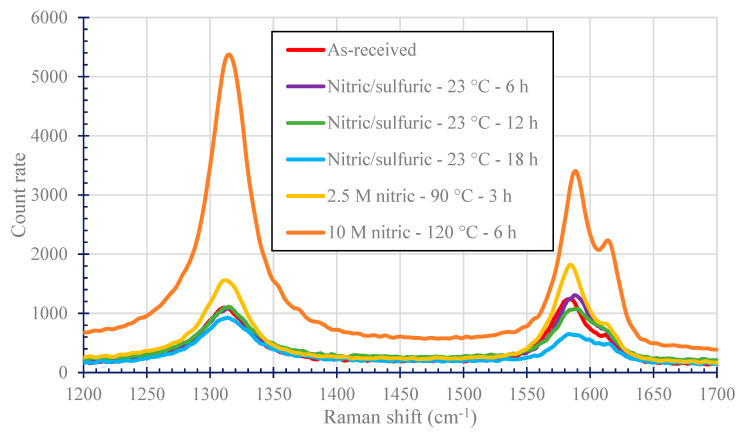
Representative Raman curves of as-received and acid-treated carbon nanotubes.

Figure 7 shows the Raman results for as-received and acid treated nanotubes. The Raman curves demonstrate distinctive peaks of the G-band at 1570 cm^−1^ and the D-band at 1350 cm^−1^. The G-band represents the sp^2^ structure of the graphitic surface, and the D-band represents disorder in the crystal structure. Figure 7 also shows the D’ band at 1620 cm^−1^, which is another indication of disorder in the graphitic structure. The D’ band becomes most pronounced for the nitric acid treatment at 120 °C for 6 h. An increased D/G ratio is evidence of a more disordered structure due to added functional groups from the acid treatment. Table 5 shows D/G ratio for the as-received and acid treated CNTs. The D/G ratio for the as-received CNT was 0.88. As expected, the D/G ratio increased the most to 1.58 (showing the highest disorder of crystalline structure likely due to the addition of oxygen containing functional groups) for the most aggressive acid treatment (10 M nitric acid at 120 °C for 6 h). As expected, higher temperatures and longer treatment times correlate with increased disordering of the graphitic structure [32,33,34,35,36].

### 3.7. XPS Results

Table 6 shows the XPS results (analyzed using CasaXPS software) from survey spectra of as-received and CNTs treated with 10 M nitric acid at 120 °C for 6 h. Table 5 shows an increase in oxygen content from 1.1 atomic% to 8.4 atomic%. It also shows the iron content decreasing from 0.4 atomic% in the as-received CNT to none present in the acid treated sample.

**Figure 8:**
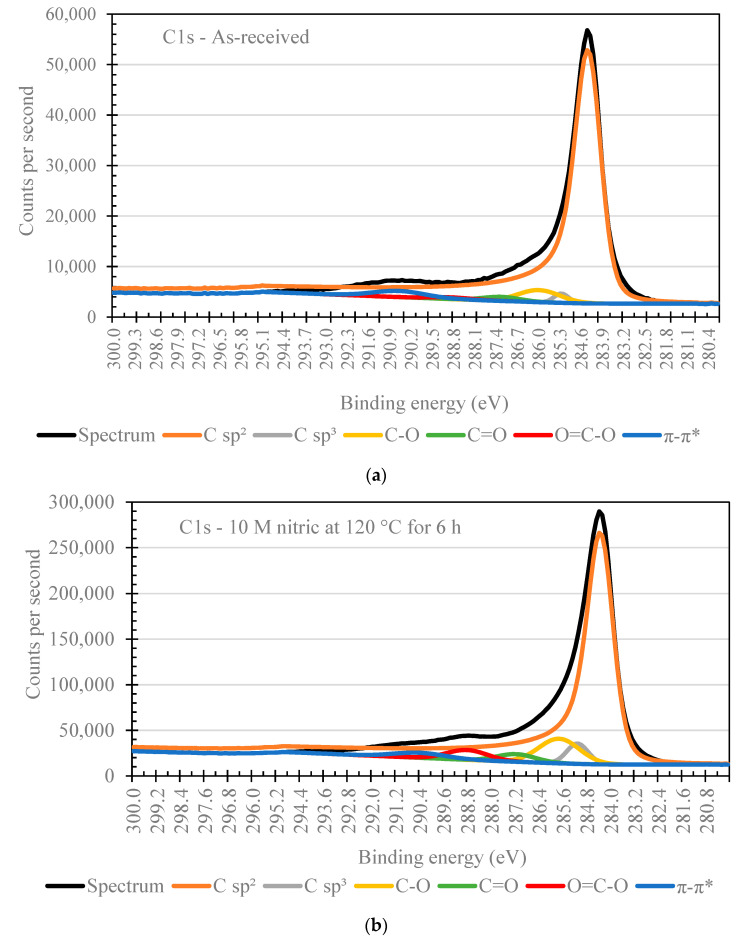
XPS C1s high resolution spectra results for (**a**) as-received and (**b**) acid treated (10 M nitric at 120 °C for 6 h) CNT.

High resolution C1s spectra were deconvoluted using CasaXPS software. A Tougaard background subtraction was used for the C1s region. Doniach Sunjic lineshape was used for sp^2^ C-C peaks. A gaussian line shape was used for peak fitting the rest of the high-resolution C1s. Figure 8 shows the high resolution C1s spectra for as-received nanotubes and nanotubes exposed to 10 M nitric acid for 6 h at 120 °C. The C1s spectra were deconvoluted into different peaks representing sp^2^ C-C at 284.5 eV, sp^3^ C-C at 285.5 eV, C–O at 286.0 eV, C=O at 287.0 eV, O=C–O at 288.5 eV, and a π-π* shake-up at 290.5 eV.

Table 7 shows the relative percentages of each functional group. As expected, the acid treated CNTs have less sp^2^ aromatic content (84.8%) than the as-received CNTs (92.5%). The oxygen containing functional groups all increased for the acid treated CNTs. For example, the C–O percentage increased from 3.2 to 6.4%. For C=O, the percentage increased from 0.4 to 2.5 %. For O=C–O, the percentage increased from 0.9 to 1.9%. These results show that the acid treatment resulted in the addition of functional groups containing oxygen (i.e., C=O, C–O, and O=C–O) and the decrease in sp^2^ character of sample surface [32,34,35,36].

## 4. Conclusions

The goal of this research was to explore the addition of a unique and commercially scalable CNT product to an aerospace grade resin. An objective of this research was to determine the effects of adding as-received MIRALON^®^ CNT pulp to CYCOM^®^ 977-3 epoxy on flexural mechanical properties and viscoelastic properties. It was determined, at the 95% confidence level, that epoxy composites containing 0.4 wt% CNT demonstrated no change in flexural modulus and a 9% increase in the flexural strength when compared to neat epoxy. Composites containing 2 wt% CNT demonstrated a 9% increase in flexural modulus when compared to neat epoxy. Composites containing 3 wt% CNT demonstrated a 16% increase in flexural modulus and a 15% decrease in flexural strength when compared to neat epoxy. Various acidic treatments were applied to the CNTs, and none of them caused an increase in flexural properties. FE-SEM micrographs appeared to show good dispersion of the as-received and treated CNTs in epoxy. FE-SEM micrographs also showed significant densification of the CNTs after acid treatment. At the 95% confidence level, DMA experiments demonstrated that epoxy composites containing ≤2 wt% as-received CNTs show no change in tanδ T_g_ when compared to neat epoxy.

The as-received CNTs and acid- treated CNTs were further characterized. TEM, TGA, and XPS results showed that the iron catalyst was removed from the as-received CNTs as a result of the 10 M 120 °C 6 h nitric acid treatment. Raman results showed that the D/G ratio increased from 0.88 for the as-received CNTs to 1.58 for the CNTs treated with 10 M nitric acid for 6 h at 120 °C. This increased D/G ratio shows an increase in the disorder (less crystalline structure) present in the acid treated CNTs. XPS results also noted a decrease in the crystalline structure for the CNTs treated with 10 M nitric acid for 6 h at 120 °C as compared to the as-received CNTs. XPS results reported that the carbon atom% decreased from 98.5 for the as-received CNTs to 91.6 for the CNTs treated with 10 M nitric acid for 6 h at 120 °C. XPS results also showed an increase in oxygen atomic% from 1.1 for the as-received CNTs to 8.4 for the CNTs treated with 10 M nitric acid for 6 h at 120 °C. Further investigation of the XPS data showed that the oxygen containing functional groups (C–O, C=O, and O=C–O) all increased for the CNTs treated with 10 M nitric acid for 6 h at 120 °C as compared to the as-received CNTs. Apparently, the 10 M nitric acid CNT treatment for 6 h at 120 °C did increase the amount of oxygen containing functional groups to the CNT surface; however, this did not translate into improved flexural properties. Per the authors’ knowledge, these material properties on as-received and acid-treated Huntsman–Merrimack MIRALON^®^ CNTs/Solvay CYCOM^®^ 977-3 epoxy have never been previously reported in the open literature.

## Figures and Tables

**Table 1:** Flexural properties (μ ± δ) of compression molded epoxy and as-received CNT/epoxy composites.

As-Received MIRALON^®^ CNTs wt%	Flexural Modulus (GPa)	Flexural Strength (MPa)	Strain (%)	*n*
0 (Vendor)	3.80 ± 0.07	144 ± 30	-	-
0	3.88 ± 0.16	136.9 ± 5.0	4.11 ± 0.24	7
0.4	3.85 ± 0.09	147.5 ± 4.0	4.27 ± 0.22	8
0.8	3.93 ± 0.08	142.9 ± 9.3	4.49 ± 0.46	7
1.5	3.93 ± 0.11	140.0 ± 4.7	4.31 ± 0.26	7
2.0	4.24 ± 0.08	141.2 ± 6.4	4.01 ± 0.24	7
3.0 ^1^	4.54 ± 0.07	116.4 ± 10.9	2.90 ± 0.30	7

^1^ Formulation dispersed without mixing cylinders.

**Table 2:** Flexural properties (μ ± δ) of compression molded epoxy, 0.4 wt% as-received CNT/epoxy, and acid treated 0.4 wt% CNT/epoxy composites.

MIRALON^®^ CNTs wt%	Flexural Modulus (GPa)	Flexural Strength (MPa)	Strain (%)	*n*
0 (Vendor)	3.80 ± 0.07	144 ± 30	-	-
0	3.88 ± 0.16	136.9 ± 5.0	4.11 ± 0.24	7
0.4 as-received	3.85 ± 0.09	147.5 ± 4.0	4.27 ± 0.22	8
0.4 acid treated (Nitric/Sulfuric—23 °C—6 h)	3.79 ± 0.11	125.2 ± 6.6	3.54 ± 0.23	10
0.4 acid treated (Nitric/Sulfuric—23 °C—12 h)	3.64 ± 0.05	124.6 ± 6.2	3.69 ± 0.27	9
0.4 acid treated (Nitric/Sulfuric—23 °C—18 h)	3.77 ± 0.07	115.1 ± 7.7	3.27 ± 0.23	9
0.4 acid treated (2.5 M Nitric—90 °C—3 h)	3.71 ± 0.07	132.7 ± 10.7	3.91 ± 0.43	8
0.4 acid treated (2.5 M Nitric—120 °C—6 h)	3.74 ± 0.06	130.2 ± 14.1	3.72 ± 0.56	7

**Table 3:** Glass transition temperatures (T_g_) (μ ± δ) as determined by DMA Q800 instrument for epoxy and CNT/epoxy composites containing up to 2.0 wt% as-received MIRALON^®^ pulp.

s-Received MIRALON^®^ CNTs wt%	tanδ T_g_ (°C)	*n*
0	249.55 ± 0.60	3
0.4	250.99 ± 0.33	3
0.8	249.42 ± 0.24	3
2.0	251.63 ± 0.32	3

**Table 4:** Iron content for CNT samples: as-received, treated with nitric/sulfuric acid mixture at 23 °C (6, 12, and 18 h), 2.5 M nitric acid at 90 °C for 3 h, and 10 M nitric acid at 120 °C for 6 h.

Sample	Iron Content (wt%)
As-received	35.2
Nitric-Sulfuric—6 h—23 °C	31.7
Nitric-Sulfuric—12 h—23 °C	31.4
Nitric-Sulfuric—18 h—23 °C	30.0
2.5 M Nitric—3 h—90 °C	26.5
10 M Nitric—6 h—120 °C	3.8

**Table 5:** Raman results for as-received and acid-treated carbon nanotubes.

Sample	D Band (Intensity)	G Band (Intensity)	D/G Ratio
As-received	1099.3	1247.2	0.88
Nitric/Sulfuric 6 h at 23 °C	1170.5	1319.7	0.89
Nitric/Sulfuric 12 h at 23 °C	1112.1	1077.3	1.03
Nitric/Sulfuric 18 h at 23 °C	1047.8	821.4	1.28
2.5 M Nitric 3 h at 90 °C	1559.1	1823.9	0.85
10 M Nitric 6 h at 120 °C	5375.4	3405.8	1.58

**Table 6:** Normalized atomic percentage from XPS results for as-received and 10 M nitric acid 120 °C 6 h CNTs.

Sample	Carbon (Atomic%)	Oxygen (Atomic%)	Iron (Atomic%)
As-received	98.5	1.1	0.4
10 M Nitric 6 h at 120 °C	91.6	8.4	~0

**Table 7:** Relative percentage of carbon states determined by deconvolution of the XPS C1s region.

Sample	C sp^2^ (%)	C sp^3^ (%)	C–O (%)	C=O (%)	O=C–O (%)	π-π* (%)
As-received	92.5	0.9	3.2	0.9	0.4	2.0
10 M Nitric 6 h at 120 °C	84.8	2.8	6.4	1.9	2.5	1.6

## Data Availability

The data presented in this study are available on request from the corresponding author. The data are not publicly available because they are currently being used for further studies.
